# Role of the Th2-like Immune Response in Obesity: IL-4 as a Metabolic Regulator and IL-13 as an Effector of Muscle Energy Metabolism

**DOI:** 10.3390/biomedicines13092208

**Published:** 2025-09-09

**Authors:** Lucía A. Méndez-García, Helena Solleiro-Villavicencio, Nallely Bueno-Hernández, Arturo Cérbulo-Vázquez, Galileo Escobedo, Marcela Esquivel-Velázquez, Miguel A. Fonseca-Sánchez

**Affiliations:** 1 Laboratorio de Inmunometabolismo, Dirección de Investigación, Hospital General de México “Dr. Eduardo Liceaga”, Ciudad de México 06720, Mexico; 2 Posgrado en Ciencias Genómicas, Universidad Autónoma de la Ciudad de México, Avenida San Lorenzo 290, Ciudad de México 03100, Mexico; helena.solleiro@uacm.edu.mx; 3 Laboratorio de Proteómica, Dirección de Investigación, Hospital General de México “Dr. Eduardo Liceaga”, Ciudad de México 06720, Mexicoesquivel.marcela@gmail.com (M.E.-V.); 4 Dirección de Investigación, Hospital General de México “Dr. Eduardo Liceaga”, Ciudad de México 06720, Mexico

**Keywords:** obesity, adipose tissue, Th2 response, IL-4, IL-13

## Abstract

The Th2 immune response, associated with allergic diseases and helminth infections, has emerged as a significant modulator of metabolic processes in adipose and liver tissues. Th2 cytokines, such as IL-4, IL-5, and IL-13, regulate energy metabolism, insulin resistance, and obesity-related issues. IL-4 and IL-13 play significant roles, while IL-5 mainly recruits eosinophils in visceral fat. IL-4 influences lipid metabolism via STAT6, promoting adipogenesis, lipolysis, and reducing leptin levels, thereby improving insulin resistance or inducing white adipose browning in the absence of leptin. IL-13 affects glucose metabolism by lowering gluconeogenesis and enhancing glucose control and increases energy expenditure in muscles during exercise via STAT3. Emerging therapies include recombinant cytokines, exosomes, and monoclonal antibodies targeting IL-4/IL-13 or IL-5, which are mostly approved for the treatment of allergic diseases. Their use in metabolic disorders is largely unexplored. Overall, Th2 cytokines are promising targets for obesity and metabolic diseases but require dedicated trials to assess benefits and risks.

## 1. Introduction

Obesity is a complex and chronic condition characterized by excessive fat deposits that can harm health. According to the World Health Organization (WHO), in 2022, about 2.5 billion adults, or 43% of the global adult population, were classified as overweight. This group comprises 890 million individuals, accounting for 16% of the worldwide adult population living with obesity [1]. However, the prevalence of overweight varies by region. In Southeast Asian and African countries, the prevalence is approximately 31%, while in countries in the Americas, it increases to as high as 67%. As a result, both overweight and obesity remain priority global public health issues. In parallel with the increase in cases of overweight and obesity, diseases such as type 2 diabetes (T2D), cardiovascular diseases (CVD), and metabolic-associated steatotic liver disease (MASLD) have also increased. The development of these diseases is associated with low-grade inflammation resulting from the accumulation of fat, which leads to widespread metabolic dysregulation [1].

White adipose tissue (WAT) is regarded as the largest endocrine organ, serving as the primary depot for fat storage. Under physiological conditions, WAT produces adipokines and cytokines. Adipokines play a crucial role in multiple processes, including insulin resistance, glucose uptake, and fatty acid oxidation, among others. Conversely, cytokines are responsible for regulating inflammation and its resolution through reparative and adaptive angiogenesis. Consequently, obesity is classified as an inflammatory immune condition. When adipose tissue expands due to excessive lipid accumulation, WAT undergoes a phenotypic transformation into characteristic, inflamed, and dysfunctional adipocytes, which subsequently leads to the infiltration of immune cells into the stroma. These dysfunctional adipocytes secrete pro-inflammatory cytokines, resulting in damage to both the adipose tissue and other organs [2].

Research on people with obesity (PWO) has shown that WAT is infiltrated by pro-inflammatory immune cells, including M1 macrophages, CD8^+^ cytotoxic T cells, Th1 cells, and MAIT cells. In contrast, adipose tissue (AT) from individuals with normal weight is characterized by the predominance of group 2 innate lymphoid cells (ILC2s), M2 macrophages, eosinophils, and Th2 cells, all of which exhibit anti-inflammatory functions [3]. Previous studies have determined the impact of physical activity and energy expenditure on the cytokine profile in individuals with obesity. In general, obesity is associated with elevated levels of cytokines originating from both Th2 and Th1 immune responses, including interleukin (IL)-5, IL-10, IL-12, IL-13, Interferon-gamma (IFN-γ), and tumor necrosis factor alpha (TNF-α). Specifically, central obesity was associated with elevated levels of IL-5, IL-10, IL-12, IL-13, and IFN-γ. Notably, low physical activity, as categorized by body mass index (BMI), was associated with an increase in Th2 response cytokines, such as IL-4 and IL-13 [4].

Th2 cells, characterized by the presence of the master transcription factor GATA3, are closely linked to type 2 immune responses. These cells promote basophilia through the production of IL-4 and IL-13, and eosinophilia through cytokines such as IL-3 and IL-5. Th2 activation also drives the development of M2 macrophages and induces B cell isotype switching, leading to the production of IgG1 and IgE. Historically, this immune profile was associated with responses to allergens and parasitic infections. However, in obesity, IL-4 and IL-13 have been found to have significant metabolic effects that are not yet fully understood. Although Th2 cells are a major source of these cytokines, they are not the only ones—other innate immune cells, such as eosinophils, basophils, and ILC2s, can also produce IL-4 and IL-13, especially in metabolically stressed tissues [5]. This review aims to clarify the role of IL-4, IL-5, and IL-13 in contributing to the immunometabolic changes observed in obesity.

## 2. Obesity: A Chronic Inflammatory… Disease?

Traditionally, obesity has been defined as a complex and chronic condition characterized by excessive adiposity that elevates health risks. Recently, a new definition has been proposed, characterizing obesity as “a disease condition that directly results from the effect of the excess adiposity on the function of organs and tissues.” Furthermore, the WHO International Classification of Diseases categorizes obesity as “a chronic complex disease,” denoting it with the code 5B81. However, historically, scoring systems utilized for the treatment of obesity have been predicated on its associated comorbidities, rather than on the clinical manifestations of obesity itself. Consequently, the absence of a definitive characterization of the disease directly induced by obesity renders the question of whether obesity can be classified as a disease highly contentious [6].

The pathophysiology of obesity arises from an imbalance between caloric intake and energy expenditure. When energy intake surpasses energy expenditure, it is stored as fat in subcutaneous adipose tissue (SAT) [7]. The brain and peripheral tissues, which include adipose tissue and the gut, serve as the primary components in this process. Energy intake elicits vagal afferent signaling and endocrine pathways that activate brain regions responsible for regulating appetite and feeding. In contrast, energy expenditure is mediated by hormonal and neurological signaling [8].

AT is categorized into functionally distinct depots: WAT is the primary lipid storage organ and exhibits significant endocrine activity. Conversely, brown adipose tissue (BAT) serves as a primary source of heat and is responsible for adaptive thermogenesis, which is stimulated by β-adrenergic activation or exposure to cold temperatures. In humans, WAT can be further subdivided into visceral white adipose tissue (VAT) and SAT, both of which are associated with the development of comorbidities [7].

Both overweight and obesity are associated with a low-grade, chronic inflammatory state. Inflammation is characterized by the infiltration of circulating pro-inflammatory macrophages (M1 or classically activated phenotype) into AT, which promotes the secretion of inflammatory cytokines such as TNF-α, IL-6, and IL-8. Meanwhile, adipocytes respond with the anti-inflammatory secretion of IL-4, IL-10, and IL-13. Adipocytes also secrete adipokines, including leptin, adiponectin, visfatin, and resistin [2]. Obesity triggers a phenotypic switch in WAT, characterized by dysfunctional and inflamed fat cells, along with the influx of immune cells. This promotes the production of local and systemic pro-inflammatory cytokines, disrupting adipose tissue and the normal functions of distant organs [9]. As a result, leptin levels increase, along with other pro-inflammatory factors, such as TNF-α, IL-1, IL-6, monocyte chemoattractant protein-1 (MCP-1), and hepatocyte growth factor (HGF). Meanwhile, levels of anti-inflammatory cytokines and adiponectin decrease. The nuclear factor kappa B (NF-κB) pathway, mediated by inhibitor of nuclear factor kappa B (IKKβ) and c-Jun N-terminal kinase (JNK)-1, is the primary driver of local and systemic inflammation induced by obesity, promoting the expression of pro-inflammatory cytokines [10]. In AT macrophages, a second pathway involving NLR family pyrin domain-containing 3 (NLRP3) regulates caspase-1 activation, leading to the release of IL-1 and IL-8, which are crucial in the development of obesity-related complications [11]. This inflammation contributes to the development of metabolic syndrome, characterized by hypertension, insulin resistance (IR), glucose intolerance, dyslipidemia, and associated CVD. Moreover, the increase in TNF-α and leptin in metabolic organs inhibits the insulin receptor, leading to T2D. Furthermore, lipid accumulation in non-adipose tissues, as well as in epicardial and perivascular tissue (EAT and PVAT), leads to hypoxia and tissue dysfunction, increasing inflammatory factors associated with CVD [12].

Gut dysbiosis constitutes an unfavorable condition frequently observed in individuals with obesity, which may induce inflammatory responses. Numerous studies comparing the gut microbiota of obese persons with that of normal-weight individuals consistently demonstrate alterations in microbial composition [13,14]. Generally, obesity has been associated with an increase in Firmicutes, including *Clostridium leptum*, *Lactobacillus reuteri*, and Veillonellaceae, as well as genera such as Fusobacteria and Roseburia. Conversely, beneficial microbes such as Akkermansia, Bacteroides, Faecalibacterium, Eggerthella, and Bacteroidetes are often reduced in obesity. These changes indicate a dysbiotic condition that could contribute to metabolic problems. Additionally, certain bacterial species have been linked to the severity of obesity and specific metabolic indicators. For example, Lactobacillus has demonstrated an inverse correlation with BMI and is positively associated with leptin levels, independent of caloric intake [14].

Furthermore, Christensenellaceae has been negatively associated with total cholesterol, triglycerides, low-density lipoprotein (LDL), and apolipoprotein B, while showing a positive relationship with high-density lipoprotein (HDL) levels. Additionally, obesity has been connected to an overgrowth of Gram-negative bacteria, resulting in elevated levels of lipopolysaccharides (LPS) within the gut, which impair its barrier function and thereby induce systemic inflammation via Toll-like receptor 4 (TLR4) signaling. This process promotes the secretion of pro-inflammatory cytokines such as TNF-α and IL-6. Conversely, short-chain fatty acids like butyrate aid in preserving gut barrier integrity and exert anti-inflammatory effects [15]; however, it remains uncertain whether they can fully counteract LPS-induced inflammation [13,14].

## 3. The Immune System and Inflammation in Obesity

### 3.1. Innate Immune Response in Obesity

Both innate and adaptive immunity play a pivotal role in the structure and homeostasis of AT. Some of the cells involved in this process are adipocytes, preadipocytes, and macrophages, which have specific functions in metabolism and inflammation [16].

In obesity, innate immunity is represented, besides macrophages, by mast cells, neutrophils, eosinophils, and dendritic cells (DCs), all of which play an essential role in contributing to IR.

In AT, leukocytes primarily consist of macrophages, which are classified into two subpopulations: M1 macrophages, predominant in obesity, express clusters of differentiation (CD) 11c, CD163, CD172, and CD44. Activated by IFN-γ and LPS, M1 macrophages cluster around apoptotic adipocytes, forming crown-like structures (CLSs), inducing aerobic glycolysis and promoting inflammation via TNF-α and IL-6 expression [17]. In lean conditions, M2 macrophages predominate, expressing arginase 1, CD206, and CD301. Induced by IL-4 and IL-13, they are uniformly distributed, secrete anti-inflammatory cytokines like IL-10, and utilize oxidative metabolism to maintain homeostasis in AT [18]. In obese mice, macrophages account for 40–60% of total immune cells in AT, whereas in lean conditions, this percentage drops to 10–15%. In vivo, the complexity increases as mixtures of M1/M2 phenotypes appear in AT with obesity in both humans and mice [19]. In the context of obesity, the recruitment of circulating macrophages into AT is facilitated by two principal mechanisms: firstly, the secretion of chemokines, including MCP-1, C-C motif chemokine ligand 2 (CCL2), and leukotriene B4 (LTB4), by adipocytes as well as the stromal vascular (SV) component of adipose tissue [20]; secondly, through signals emanating from dying and stressed cells that are recognized by the NLRP3 and TLR-4, two pattern-recognition receptors (PRRs) on macrophages. Upon recruitment, adipose tissue also conveys retention signals, such as those governed by netrin-1 [21].

New technologies, such as single-cell sequencing, have identified additional AT macrophage phenotypes. For instance, a recently reported subtype in adipose tissue, called M3, is localized in CLS and expresses the chemokine receptor (CCR)7. Additionally, another type, metabolically activated macrophages (MMe), is induced by high glucose, insulin, and palmitate levels, resulting in overexpression of ATP-binding cassette transporter (ABCA1) and the CD36 marker. This suggests that obesity-induced inflammation may influence macrophage polarization in AT [20].

Obese mice have more mast cells than controls, and their deficiency correlates with IR [22]. Mast cells regulate thermogenesis in adipose tissue by genetically removing tryptophan hydroxylase 1 (*Tph1*), which increases WAT mitochondrial uncoupling protein 1 (Ucp-1), protecting mice from obesity and IR [23]. Neutrophils contribute to obesity by producing elastase and releasing pro-inflammatory cytokines that recruit and activate T lymphocytes at inflammation sites. Although macrophages are the primary cells studied in inflammation due to fat accumulation, neutrophils are the first immune cells that infiltrate adipose tissue during high-fat diet (HFD)-induced obesity, attracted by IL-8. A key function of neutrophils is the formation of neutrophil extracellular traps (NETs). In humans, obesity-related inflammation correlates with high NET levels [24]. In a diet-induced obesity (DIO) mouse model, NET formation plays a crucial role in the inflammatory response by regulating the expression of MCP-1, as measured by cathelicidin-related antimicrobial peptide (CRAMP), highlighting the importance of NETs in obesity-induced inflammation [25].

In contrast, eosinophils produce Th2-like responses (IL-4 and IL-13) in VAT, resulting in the differentiation of macrophages into M2 cells. ILC2s support eosinophils and their anti-inflammatory response. In animal models, eosinophil deficiency in AT causes weight gain, decreased insulin sensitivity, and glucose intolerance compared to wild-type animals. Like mast cells, eosinophils protect against obesity by regulating thermogenesis through the actions of IL-4 and IL-13. The role of eosinophils in obesity is established in mice, but the results in humans regarding eosinophils in AT homeostasis are controversial. Eosinophilia is associated with a decreased risk of developing T2D, but it also correlates with BMI. A high eosinophil count is linked to metabolic syndrome. Depleting eosinophils with anti-IL-5 therapy reduces BMI in patients with obesity and severe asthma. More studies are needed to clarify the role of eosinophils in human AT [26].

In AT, DCs differentiate in the first three days of HFD, and after seven days of this diet, the DC population significantly increases in VAT and SAT. In VAT, the infiltration rate of DCs increases in response to chronic inflammation and remains sustained until 8 weeks on an HFD; CCR7 regulates their migration. After 16 weeks of HFD, the DCs’ percentage diminishes in VAT but not on SAT, suggesting that DCs’ expansion depends on a specific depot in AT [27]. A prolonged HFD considerably alters the global transcriptional profile of mouse splenic DCs (SpDCs) in genes involved in lipid metabolism and mitochondrial function by increased fatty acid oxidation (FAO), leading to intracellular accumulation of reactive oxygen species (ROS), inhibiting the antigen (Ag) presentation to T cells, which is reversed by the treatment with antioxidants [28].

This evidence demonstrates that innate immunity plays a crucial role in regulating AT dynamics, function, and inflammation, particularly in the context of obesity. It contributes to obesity-related metabolic dysfunctions and provides opportunities to identify therapeutic targets for reducing inflammation and improving affected metabolic parameters.

### 3.2. Adaptive Immune Response in Obesity

Traditionally, obesity-related inflammation has been focused on macrophages; however, the adaptive immune system also plays a crucial role in regulating inflammation in obesity. Lymphocytes make up 10% of non-adipocyte cells in adipose tissue, including B and T cells [29].

B cells play a crucial role in IR. In DIO mice, obesity promotes B cell infiltration into the VAT, primarily comprising class-switched, mature IgG^+^ B cells. While the absence of B cells protects from disease, it does not prevent weight gain. Pathogenic IgG antibodies link B cells to glucose metabolism. In PWO, the IR correlates with a specific IgG autoantibody profile [30]. Recent findings suggest that obesity triggers a dysfunctional B cell response similar to that observed in aging, which some individuals may experience after weight loss [31]. However, further research is required to elucidate the underlying mechanisms.

In murine obesity models, CD8^+^ T cells in adipose tissue increase after two weeks of HFD and remain elevated until week 15, along with higher IFN-γ and granzyme B levels. This recruitment is driven by macrophage inflammatory protein-1α (MIP-1α) and chemokine receptors CCR1, CCR4, and CCR5. In both murine and human subjects, tissue-resident CD8^+^ T cells in adipose tissue contribute to T2D and IR through the secretion of IFN-γ and TNF-α. Furthermore, these cells also exhibit high proliferation and resistance to cytokine-mediated suppression, clarifying their role in obesity-induced insulin resistance and diabetes, as well as their link to conditions such as MASLD and obesity-related cancers [32].

Concerning CD4^+^ T cells, observations in obese animal models indicate an increase in the production of IFN-γ by Th1 cells, which subsequently leads to IR. Conversely, research demonstrates that the deletion of IFN-γ exacerbates obesity-induced IR [33]. In VAT from PWO, infiltrating T cells exhibit a heightened polarization towards Th1 cells, type 1 CD8^+^ T cells (Tc1), and T1-like CD4^+^CD8^+^ T cells. These cells express programmed cell death protein-1 (PD-1), which IFN-γ induces, and their activation shows a negative correlation with IR [34]. Among other pro-inflammatory responses, Th17 cells secrete mainly IL-17, which, in vitro, inhibits adipocyte differentiation. However, in mice, the Th17 response did not appear to play a causal role in IR [35]. In contrast, this response seems to be related to T2D; in humans, IL-17 is associated with the severity of this disease [36]. Furthermore, a meta-analysis reveals an imbalance between Th17 and Treg proportions, which favors a pro-inflammatory environment in patients with T2D [37].

Tregs and the Th2 response mediate the anti-inflammatory activity of CD4^+^ T cells. In obesity, Treg proportions decrease in VAT, where they are attracted to CLRs associated with macrophages and lymphoid cells [38]. Despite the known roles of Tregs in obesity-related inflammation and IR, studies yield contradictory results. While IL-2-induced Tregs are reported to improve IR, other studies have failed to confirm this, suggesting instead that Tregs may promote IR [22].

Another anti-inflammatory response of adaptive immunity is the Th2 response, which is typically associated with respiratory diseases, such as asthma, and infections caused by parasites and helminths. As a result, research on this response in metabolic diseases and obesity has been limited. However, recent studies have shown that the Th2-like immune response plays an active role in these conditions, making it increasingly relevant. The following sections will examine the current findings on the role of the Th2 response in obesity.

#### Th2 Cell Differentiation and Its Central Role in Type 2 Cytokine Regulation

Upon the immunological synapse, CD4^+^ T cells initiate transcriptional programs that guide their differentiation into specialized effector subsets. The strength and duration of T-cell receptor (TCR) signaling, along with antigen affinity and dwell time, critically influence lineage commitment, including the fate of Th2 cells [39]. T effector CD4^+^ cells (Teff2 cells) that secrete type 2 cytokines are primed in lymphoid organs, with their differentiation occurring in tissues. Research has focused on identifying antigen-presenting cells (APCs) that promote the Th2 response. It is established that CD301b^+^ DCs migrate to the lymph node via CCR8 in response to allergens, facilitating Teff2 cell priming. Once primed, Teff2 cells move to non-lymphoid tissues, where tissue-specific mechanisms regulate their differentiation. The signals that control Th2 cell function in non-lymphoid tissues remain unknown [40]. The prevailing model of Th2 cell subset differentiation emphasizes the critical role of the cytokine environment in determining CD4^+^ T cell fate. In this context, the differentiation of Th2 cells begins with the binding of IL-4 or IL-13 to the IL-4 receptor (IL-4Rα), which induces the phosphorylation of STAT6. STAT6 then dimerizes and translocates to the nucleus, promoting the expression of GATA-3 (a key Th2 lineage-determining factor), which in turn enhances the production of type 2 cytokines [41].

GATA-3 can self-activate, thereby stabilizing its expression and maintaining the production of IL-4 and IL-13. Studies show that this activation is unnecessary in early Th2 cell differentiation but essential later. Thus, several non-canonical signaling pathways exist for activating IL-4 and IL-13, independent of IL-4Rα and STAT6, such as those regulated by IL-2, STAT5A, and the Notch pathway [42].

Other immune cells, like basophils, eosinophils, and mast cells, constitutively express IL-4 and IL-13, serving as early sources of type 2 cytokines during allergic responses. These cells also play a significant role beyond allergies and helminth infections. Specifically, IL-4 production by eosinophils is crucial for regulating metabolic pathways, including brown fat formation, macrophage homeostasis, and glucose tolerance in HFD-fed animal models [43]. Additionally, IL-13 has recently been recognized for its role in metabolic homeostasis, influencing glucose metabolism, energy expenditure, and inflammatory responses [44] as discussed in the following sections.

## 4. IL-4 as a Metabolic Modulator

**Figure 1:**
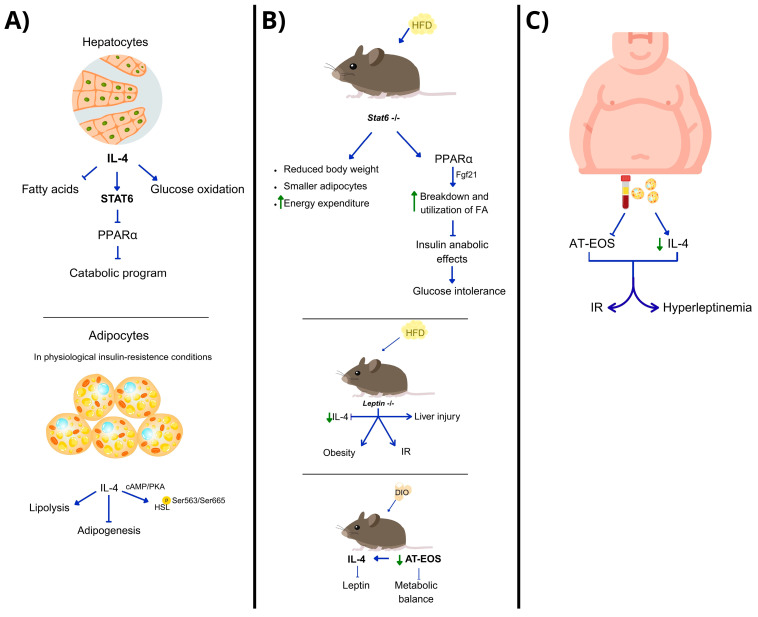
Studies determining the role of IL-4 in metabolism. (**A**) In hepatocytes, IL-4, via STAT6, regulates glucose metabolism by inhibiting PPAR-α and its metabolic program, while in adipocytes, it promotes lipase phosphorylation to inhibit lipolysis and adipogenesis during physiological insulin resistance. (**B**) Animal studies on high-fat diets show that IL-4 signals through STAT6; its deletion activates PPAR-α, which promotes fatty acid utilization and breakdown. In leptin-deficient conditions, decreased IL-4 levels lead to insulin resistance and obesity. In diet-induced obesity (DIO) models, IL-4 inhibits leptin, improving IR. This model also shows a decrease in AT-EOS, leading to metabolic balance disruption. (**C**) Obesity levels in humans inversely correlate with IL-4 levels and eosinophil numbers in adipose tissue and serum, which contribute to insulin resistance and hyperleptidemia.

IL-4 influences metabolic processes in the liver and WAT through the Signal transducer and activator of transcription (STAT)-6 pathway, underscoring its therapeutic potential. In hepatocytes, IL-4 treatment suppresses fatty acids (FAs) and promotes hepatic glucose oxidation, an effect reversed in STAT6-deficient mice that show increased FAO and peroxisome proliferator-activated receptor-alpha (PPARα) activity [45]. IL-4 activates STAT6, which trans-represses PPARα, inhibiting its catabolic program (Figure 1A). In an HFD context, STAT6-null mice have reduced body weight and smaller adipocytes due to an inability to undergo hypertrophic changes and increased energy expenditure. The absence of STAT6 enhances PPARα transcriptional activity compared to lean animals, promoting FA breakdown regulated by fibroblast growth factor 21 (Fgf21) (Figure 1B). Additionally, increased PPARα activity resulting from STAT6 silencing inhibits insulin’s anabolic effects, leading to glucose intolerance. STAT6-null livers show IR, indicated by decreased protein kinase B (AKT) pathway phosphorylation [45].

IL-4 not only has anti-inflammatory properties but also regulates energy homeostasis and metabolism. IL-4 and IL-4R are associated with metabolic parameters, such as HDL levels and T2D. In adipose tissues, IL-4 enhances insulin sensitivity and glucose tolerance, while inhibiting adipogenesis and lipid accumulation, and regulates adipose-derived adipokines essential for maintaining energy balance [46]. IL-4’s mechanisms for inhibiting adipogenesis involve activating hormone-sensitive lipase (HSL) [47] and promoting lipolysis in mature adipocytes [48] (Figure 1A). Studies show that IL-4 enhances HSL phosphorylation at Ser563/Ser665 via the cyclic adenosine monophosphate/protein kinase A (cAMP/PKA) pathway, thereby increasing lipolytic activity, as measured by glycerol release from triacylglycerol processing. In vivo, IL-4 induces hepatic Ser563 phosphorylation but not in AT. Six weeks of IL-4 treatment in mice with HFD led to significantly lower body weight. However, IL-4 stimulates lipolysis in chow-fed mice only, indicating that its metabolic regulatory effect is limited to insulin-sensitive conditions. Once IR develops, IL-4 is insufficient to improve energy homeostasis [48].

In leptin-deficient or leptin-resistant HFD models, circulating levels of IL-4 decrease, leading to metabolic abnormalities like obesity, hyperglycemia, IR, liver injury, and changes in AKT, STAT3, and STAT6 pathways. Supplementation with IL-4 improves obesity, restores molecular pathways, and promotes browning of white adipocytes in epididymal tissue and a 3T3-L1 cell line [49] (Figure 1B). However, DIO diet mice show reduced leptin via IL-4, improving IR. In this model, a notable decrease in resident AT eosinophils (AT-EOS), which produce IL-4, disrupts metabolic balance. Similarly, obese individuals have fewer AT-EOS and lower IL-4 levels in plasma and AT compared to lean counterparts, correlating with the presence of IR and hyperleptinemia (Figure 1C). Ex vivo and in vitro studies show that IL-4 or eosinophil exposure results in reduced leptin production by human adipocytes. These findings suggest that IL-4 serves as a metabolic regulator, linking eosinophils, AT, and leptin levels, making it a potential therapeutic candidate for obesity and its complications by regulating hyperleptinemia [50]. Table 1 summarizes the established mechanisms regulated by IL-4 in liver and adipose tissue metabolism, as well as their therapeutic implications.

## 5. IL-5 Coordinates the Eosinophil Distribution

In VAT, eosinophils play a crucial role in maintaining metabolic homeostasis and activating alternatively activated macrophages (AAMs). IL-5 regulates the release of eosinophils from the bone marrow and their tissue distribution [51]. IL-5-deficient animal models exhibit reduced energy expenditure, increased adiposity, and metabolic impairment, accompanied by decreased eosinophil levels in VAT without affecting systemic pools. This suggests that eosinophil recruitment can occur independently of IL-5 involvement. In this context, IL-13 activates eotaxins, while IL-4 enhances endothelial cell integrins, aiding eosinophil recruitment. In VAT, ILC2s are the primary producers of IL-5 and IL-13.

Functionally, ILC2s resemble CD4^+^ Th2 cells and are widespread in tissues without antigen stimulation. In obese mice, exogenous IL-33 administration promotes the Th2 response, improving insulin sensitivity and increasing CD25 expression in ILC2s, indicating activation and leading to eosinophil and AAM accumulation in VAT [52]. Additionally, CD300f, an Ig superfamily receptor, regulates IL-5 in eosinophil accumulation and IL-4 production in adipose tissue by managing the extracellular signaling-regulated kinase (ERK) and Akt signaling pathways [53]. IL-5 not only functions as the main growth factor for eosinophils, but in a severe asthma model, it was observed to enhance the pro-fibrotic roles of eosinophils through the transforming growth factor beta (TGF-β) pathway. Therefore, IL-5 could serve as a therapeutic target for modulating eosinophil-regulated molecular mechanisms not only in the airways [54] as is shown in Table 2.

## 6. IL-13 in Metabolic Homeostasis and Energy Expenditure

**Figure 2:**
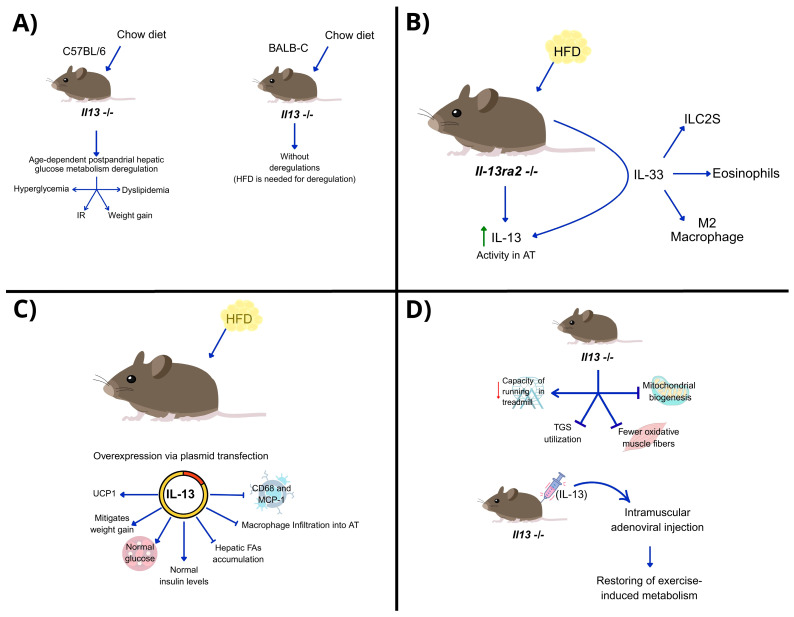
Animal models show the role of IL-13 in metabolism and energy expenditure. (**A**) 1) *Il13^-/-^* animals on a standard diet experience age-dependent postprandial liver glucose metabolism disruption leading to hyperglycemia, IR, weight gain, and dyslipidemia. However, this effect varies by strain: while C57BL/6 mice are more prone to metabolic diseases, BALB/c mice, which are more resistant, need a high-fat diet (HFD) to show similar effects from IL-13 deficiency. (**B**) In HFD-fed animals lacking IL-13Ra2, a decoy receptor that blocks IL-13, elevated IL-13 levels are seen due to the action of the alarmin IL-33. This results in increased M2 macrophages, eosinophils, and expanded ILC2S. (**C**) In HFD-fed mice, overexpression of IL-13 boosts UCP-1 levels (a protein linked to energy metabolism), prevents excessive weight gain, promotes glucose balance and normal insulin levels, inhibits hepatic FA accumulation, and reduces macrophage infiltration into adipose tissue, along with pro-inflammatory markers like CD68 and MCP-1. (**D**) IL-13-deficient animals undergoing endurance exercise show reduced treadmill performance, impaired triglyceride use, decreased oxidative muscle fibers, and mitochondrial biogenesis—effects that reverse with intramuscular IL-13 administration.

Over the past decade, studies have confirmed the role of IL-13 in metabolism, particularly glucose homeostasis and gluconeogenesis (Table 3). Young C57BL/6 mice, genetically deficient in *IL-13* and fed a chow diet, exhibit age-dependent postprandial hepatic glucose metabolism deregulation, without increased pro-inflammatory cytokines. These disturbances lead to hyperglycemia, IR, weight gain, and dyslipidemia, resulting in metabolic syndrome. In contrast, BALB/c mice, which are less susceptible to metabolic diseases, do not show deregulation in hepatic glucose levels with *IL13* deletion alone; an HFD is needed for metabolic disruption (Figure 2A). Thus, IL-13 is crucial when insulin levels are overwhelmed, such as with aging or overnutrition [55]. In hepatocytes, exogenous IL-13 induces the phosphorylation of STAT3, which suppresses the expression of gluconeogenic genes and thereby reduces glucose production. This activity may be crucial in mice during the postprandial period, when glucose availability can lead to hyperglycemia, highlighting the key role of IL-13 in regulating glucose metabolism via STAT3 [56].

In obese mice under HFD, IL-13Ra2 functions as an IL-13 decoy receptor, neutralizing the cytokine. Mice lacking IL-13Ra2 show improved metabolic outcomes from HFD and increased IL-13 activity in AT. The concentration of IL-13 is regulated by IL-33, which increases serum and tissue IL-13 levels and promotes the expansion of ILC2s, eosinophils, and anti-inflammatory macrophages. IL-13 mediates IL-33-driven metabolic homeostasis on HFD, affecting body weight and fasting blood glucose, but IL-13Ra2 deletion alone does not alter HFD’s metabolic effects [57] (Figure 2B).

IL-13 is a crucial cytokine in regulating energy expenditure in muscle tissue, especially during endurance exercise. In animal models subjected to HFD with IL-13 overexpression via plasmid transfection, weight gain was mitigated without changing food intake. These mice maintained normal glucose and insulin levels while showing reduced hepatic FA accumulation. IL-13 induction also inhibited macrophage infiltration into the AT and suppressed the expression of inflammatory molecules, such as CD68 and MCP-1 (Figure 2C). This intervention upregulated *Ucp1*, a gene associated with energy expenditure, suggesting that IL-13 plays a role in thermogenesis [58].

Knudsen and collaborators found that endurance exercise increases plasmatic IL-13 levels in mice and humans by expanding ILC2s in muscles. *Il13*-deficient mice had reduced running capacity, impaired triglyceride utilization, and mitochondrial biogenesis, indicating an *Il13*-dependent gene signature for fatty acid metabolism. These mice also had fewer oxidative muscle fibers and lower endurance and glucose tolerance compared to wild-type controls. Intramuscular adenoviral injection of IL-13 reversed these effects, restoring metabolism during exercise [44] (Figure 2D). These findings highlight the role of IL-13 in exercise-induced adaptations and metabolic reprogramming in muscle tissue, mediated through IL-13 receptor signaling and STAT3 activation, similar to its regulation of gluconeogenesis in the liver via STAT3 phosphorylation.

IL-13, mediated by STAT6, is not expressed in skeletal muscle, indicating the presence of alternative signaling pathways in this tissue [44]. This suggests that the IL-13/STAT3 axis may be functional in muscle, liver, and potentially other tissues. Notably, IL-13 overexpression in muscle enhances the expression of the Estrogen-Related Receptor (ERR) α and γ (Esrra and Esrrg), which are vital regulators of metabolic transcriptional programs [44]. However, it is unclear if these mechanisms are similarly regulated in human muscle during physical activity [59].

In obesity, chronic activation of the JAK-STAT3-SOCS3 signaling pathway by leptin and IL-6 has been described [59]. However, a direct link between IL-13 and this pathway in adipose tissue has not been established.

IL-13 has also been linked to metabolic regulation through the adipokine growth differentiation factor 15 (GDF15). In mouse studies, IL-13 increased GDF15 levels in adipose tissue and plasma in a STAT6-dependent way. Recombinant IL-13 improved glucose tolerance in HFD mice, but only when GDF15 was present. GDF15 is a member of the TGF-β family, regulating appetite through the GDNF Family Receptor Alpha-Like (GFRAL) and promoting lipid oxidation and lipolysis in peripheral tissues. These results suggest that GDF15 is a key adipokine that can be influenced by IL-13, potentially having beneficial effects on glucose metabolism [60].

It is hypothesized that the Th2 immune response evolved to modulate adaptive responses to physiological stressors, such as pathogen infection, tissue damage, IR, and endurance exercise; however, this remains largely untested. It is still unclear whether these adaptive functions are compromised in obesity and its associated low-grade chronic inflammation [61]. Understanding the IL-13/STAT3 axis under these conditions may offer new insights into Th2 immunometabolic adaptations.

## 7. Th2 Response in Obesity and Asthma

Chronic asthma is a complex and diverse condition marked by excessive mucus production, abnormal narrowing of the airways, and ongoing lung inflammation. It is driven by a dominant Th2 helper immune response, along with related cytokines, and the buildup of eosinophils whose survival is fueled by IL-5. These eosinophils, in turn, produce specific antigens triggered by IL-4 and IL-13, which increase muscle sensitivity [62].

Researchers have identified a phenotype called “obesity-associated asthma,” which is particularly common in women. This condition is characterized by severe symptoms that typically develop later in life and are more challenging to manage. Unlike traditional asthma, which is triggered by allergens and driven by a Th2 inflammatory response, obesity-associated asthma is linked to irritants like respiratory diseases, tobacco smoke, and obesity. This type of asthma does not involve a Th2 response [63].

Recent studies have explored the connection between Th2 response cytokines and NLRP3 in human and mouse models of obesity-induced asthma. In people with asthma, researchers found a link between BMI and the inflammasome response, with increased levels of IL-5 and IL-13. In animal models, obesity and asthma induced by a high-fat diet were associated with an increased NLRP3 inflammasome response and eosinophil-mediated inflammation in the airways. When the inflammasome was inhibited, airway hyperresponsiveness decreased, but IL-5 and IL-13 levels remained unchanged. In contrast, inhibiting these cytokines reduced hyperresponsiveness and the NLRP3 inflammasome response in mouse models of obesity and asthma. These findings suggest that the Th2 response plays a role in obesity-related asthma through a complex mechanism regulated by NLRP3. Therefore, studying interventions that inhibit IL-5 and IL-13 could be a promising therapeutic approach for individuals with these conditions [64].

## 8. Emerging Drugs and Biologic Therapies Regulating Th2 Cytokine Activity in Obesity

As discussed, Th2 cytokines, particularly IL-4, IL-5, and IL-13, have diverse immunometabolic functions that impact adipose tissue inflammation, energy regulation, and overall metabolic balance. Initial research and clinical studies are investigating the pharmacological modulation of these compounds as potential treatments for obesity and related metabolic conditions.

The available evidence is limited, particularly for IL-13, which is scarce in the context of obesity. Therefore, IL-13 is not covered as a separate section in this article. However, due to its shared receptor pathways and functional similarities with IL-4, it warrants further exploration as a potential therapeutic target in obesity. The following subsections review the existing data on IL-4 and IL-5, combining preclinical and clinical evidence, with a focus on mechanistic insights while acknowledging the current limitations in translation (Table 4).

### 8.1. IL-4-Based Therapeutic Approaches

Recent experimental studies have investigated the role of IL-4 as a modulator of key metabolic and inflammatory pathways involved in the development of obesity. Phu et al. [65] found that in vitro, exosomes from THP-1 macrophages polarized with IL-4 (THP-1-IL-4-exo) promoted an anti-inflammatory phenotype in primary macrophages promoted an anti-inflammatory M2-like phenotype in primary macrophages, upregulated microRNAs involved in energy metabolism and adipose tissue remodeling (miR-21, miR-99a, miR-146b, miR-378a), and downregulated miR-33, a negative regulator of fatty acid oxidation and lipolysis. In adipocytes, these exosomes enhanced insulin-dependent glucose uptake via PPARγ, increased *UCP1* expression, boosted mitochondrial activity, and stimulated lipophagy. In obese wild-type and *Ldlr^−/−^* mice, intraperitoneal injection of THP-1-IL-4-exo reduced inflammatory signaling, attenuated hepatic steatosis, promoted WAT browning, and improved glucose tolerance [65].

IL-4 has also been identified as playing a critical role in the context of a rodent *Fas*-mutant MRL/lpr model, characterized by animals that display reduced adiposity and increased thermogenic activity compared to MRL/MpJ controls [66]. Animals with an HFD and a *Fas* mutation did not develop diet-induced obesity and exhibited a decreased WAT M1:M2 ratio, indicating a shift toward the M2 phenotype. In these *Fas*-mutant mice, WAT showed higher expression levels of *Ucp1*, *IL4*, *IL10*, and tyrosine hydroxylase during HFD. Additionally, cold exposure further increased *Ucp1* expression and browning markers in these animals. The authors suggest that these effects may involve STAT6 activation and IL-4/IL-10/IL-10-driven M2 polarization [66].

In addition, central effects of IL-4 on appetite regulation and energy balance have been investigated. In a leptin receptor-dysfunctional mouse model (*Leptin*^145E/145E^), IL-4 administration reduced body weight, food intake, and blood glucose, while downregulating orexigenic (agouti-related peptide and neuropeptide Y) and upregulating anorexigenic (proopiomelanocortin) neuropeptides in the hypothalamus. These changes were accompanied by decreased hypothalamic inflammation, improved insulin–AKT and JAK–STAT signaling, enhanced insulin sensitivity, and increased *Ucp1* expression in white adipose tissue, indicating coordinated central and peripheral metabolic effects [67].

Although IL-4 and IL-13 share receptor components and overlapping signaling pathways, their roles in obesity-related adipose tissue fibrosis are still unclear. In an ex vivo WAT culture model, both cytokines were found to induce fibrosis-related gene and protein expression through IL-4R and macrophage-dependent mechanisms; this was partly confirmed in vivo [68]. While WAT fibrosis is associated with AT dysfunction in obesity, the direct contribution of IL-4 to this process has not yet been definitively established.

### 8.2. IL-5-Based Therapeutic Approaches

As we have reviewed, IL-5 plays a central role in the survival, proliferation, and activation of eosinophils, which have emerged as key immune cells in the regulation of AT homeostasis. However, there is limited and mostly indirect clinical evidence connecting IL-5 modulation to metabolic outcomes, mainly derived from studies on adults with severe asthma treated with anti-IL-5 biologics [69]. For example, a retrospective study involving 51 patients treated for several months with mepolizumab, reslizumab, or benralizumab showed an average decrease in BMI of 1 point across the cohort, with a higher reduction of up to 1.7 points in those with obesity (BMI > 30). Nonetheless, the study highlights confounding factors—such as long-term corticosteroid use and asthma-related inflammation—that limit its direct applicability to obesity.

Another clinical study observed similar BMI changes in patients receiving anti-IL-5 therapy, but no consistent association with agents was found [70]. These results suggest that eosinophil depletion may impact body composition in some instances, although the metabolic implications require further investigation.

Preclinical studies have indicated a potential beneficial effect of IL-5-driven eosinophil activity. In research by Rozenberg et al. [53], *IL-5 Tg/Cd300f*^−/−^ mice fed with an HFD showed increased eosinophil counts and IL-4 levels in the AT, accompanied by a M2 macrophage accumulation, less weight gain, and better glucose tolerance. Likewise, the deletion of the inhibitory receptor CD300f boosted eosinophil recruitment, M2 macrophage polarization, and offered protection against diet-induced obesity [53].

Together, these studies suggest that IL-5 is a promising yet under-investigated therapeutic target for obesity and metabolic diseases, primarily through approaches that enhance eosinophil activity rather than reduce it.

Table 5 offers a succinct overview of the primary biologics and experimental strategies targeting the IL-4, IL-5, and IL-13 pathways. The table delineates their molecular targets, clinical status, and current evidence regarding metabolic outcomes. Although several agents have already received approval for use in allergic and inflammatory conditions, no clinical trials have been conducted to date to evaluate their efficacy in obesity or related metabolic disorders.

## 9. Discussion

Traditionally, the Th2 immune response was associated with allergic reactions and parasitic infections; however, in recent years, the Th2 response has emerged as a significant modulator of energy metabolism and adipose tissue function. Among its cytokines, IL-4, IL-5, and IL-13 have shown distinct but complementary roles in regulating metabolic homeostasis, insulin sensitivity, and inflammation.

IL-4 stands out as an immunometabolic regulator, capable of inhibiting adipogenesis and promoting lipolysis in insulin-sensitive conditions, while also modulating leptin signaling and inducing the browning of WAT in leptin-deficient models. IL-5 primarily contributes to modulating eosinophil activity, thereby supporting anti-inflammatory responses in VAT. IL-13, meanwhile, plays a broader role in regulating glucose metabolism and energy expenditure, particularly under metabolic stress, such as high-fat diets or aging, through STAT3 signaling.

Preclinical and early clinical research has explored pharmacological strategies to modulate IL-4 and IL-5 in obesity and metabolic dysfunction. In animals, IL-4-based interventions—like cytokine delivery and IL-4-polarized exosomes—show coordinated central and peripheral effects, including hypothalamic appetite regulation, improved glucose homeostasis, browning of WAT, and tissue inflammation reduction. In humans, anti-IL-5 therapies, mainly studied in severe asthma, may affect BMI, but confounding factors like corticosteroid use limit interpretation. Preclinical findings reinforce the metabolic role through eosinophil-maintained M2 macrophages and the promotion of thermogenesis. As shown in Table 5, currently available IL-4-, IL-5-, and IL-13-targeted therapies underscore the translational gap between the established indication in allergic diseases and the lack of clinical evidence in obesity.

Although pharmacological modulation of Th2 cytokines in obesity is still in its early stages, recent studies suggest a translational potential. Future research should focus on clinical trials evaluating metabolic endpoints in patients receiving IL-4-, IL-5-, or IL-13-targeted therapies. Additional efforts could optimize Th2 immune activities, like adipose eosinophilia and WAT browning, while reducing pro-fibrotic or adverse effects. Using network pharmacology, longitudinal immunometabolic profiling, and precision medicine may help identify patient groups most likely to benefit from Th2-targeted treatments.

## Figures and Tables

**Table 1:** IL-4 in hepatic and adipose tissue metabolism: mechanisms and therapeutic implications.

Area	Pathways Involved	Therapeutic Implications	Ref.
Liver (hepatocytes)	IL-4 → STAT6 → PPARα trans-repression	IL-4 may regulate hepatic metabolism; STAT6 is key for balance between lipid and glucose metabolism.	[45]
White adipose tissue (WAT)/adipocytes	IL-4→ STAT6 → ↓ adipogenesis IL-4 → HSL activation via cAMP/PKA	IL-4 enhances lipid breakdown in WAT; potential in early intervention before IR develops.	[46,47,48]
HFD/DIO-leptin-deficient models and 3T3-L1 cell line	↓ IL-4 → obesity, hyperglycemia, IR IL-4 deficiency → ↓ AT-EOS IL-4 supplementation restores STAT3/STAT6/AKT	IL-4 restores metabolic balance in obesity and leads to browning of white adipocytes.	[49]
Human adipocytes (ex vivo or in vitro)	IL-4 regulates leptin via eosinophils	IL-4 could regulate hyperleptinemia and improve outcomes in obesity.	[50]

Arrows ‘→’ mean ‘leads to’ while arrows ‘↓’ mean ‘decrease’.

**Table 2:** IL-5-dependent and -independent mechanisms controlling eosinophil recruitment and function.

Area	Pathways Involved	Therapeutic Implications	Ref.
Visceral Adipose Tissue (VAT)	IL-5 → eosinophil release and tissue distribution	IL-5 is essential for VAT eosinophil homeostasis, impacting metabolic regulation.	[51]
Eosinophil recruitment	IL-13 eotaxins IL-4 endothelial integrins	Redundant mechanisms support eosinophil recruitment; IL-5 is critical but not exclusive.	[51]
Innate lymphoid cells type 2 (ILC2s)	IL-33 → ILC2 activation → IL5/IL13 production	Targeting ILC2 activation via IL-33 may enhance VAT immune–metabolic function.	[52]
Regulation by CD300f receptor	CD300f → ERK/Akt signaling → IL-5 and IL-4 modulation	Modulating CD300f may influence local immune–metabolic function.	[53]
Fibrosis and asthma models	IL-5 → TGF-β pathway → profibrotic eosinophil activity	IL-5 may be a therapeutic target in fibrotic and inflammatory diseases.	[54]

Arrows ‘→’ mean ‘leads to’.

**Table 3:** Regulatory functions of IL-13 in glucose metabolism, energy expenditure, and immune modulation.

Area	Pathway Involved	Therapeutic Implications	Ref.
Liver (hepatocytes)	IL-13 → STAT3 phosphorylation → ↓ gluconeogenesis	IL-13 regulates glucose metabolism via STAT3, especially under metabolic stress.	[56]
Adipose tissue (AT)	IL-13Ra2 → ↓ IL-13 activity IL-33 → ↑ IL-13 and anti-inflammatory immune cells	Blocking IL-13Ra2 may restore IL-13 function in obesity. IL-13 reduces adipose inflammation.	[57]
Skeletal muscle	IL-13 → ↑ UCp1, ERRα and ERRγ expression → ↑ thermogenesis and FA metabolism	IL-13 supports muscle metabolic reprogramming and exercise adaptation via non-STAT6 signaling.	[58,59]
Exercise-induced adaptation	Endurance exercise → ↑ILC2 → IL-13/STAT3 activation	IL-13 may mediate physiological adaptation to exercise and stress; potential therapeutic target for metabolic muscle disorders.	[44]

Arrows ‘→’, ‘↓’ and ‘↑’ mean ‘leads to’, ‘decrease’ and ‘increase’ respectively.

**Table 4:** Research on IL-4 and IL-5 in obesity and metabolic regulation.

Cytokine	Model/Study Design	Main Findings	Ref.
IL-4	In vitro (THP-1 macrophages, 3T3-L1 adipocytes) + in vivo (mice, HFD, WT, *Ldlr*^−/−^)	IL-4-polarized (M2-like) macrophage exosomes increased anti-inflammatory miRNAs, reduced miR-33, enhanced GLUT4, UCP1, OXPHOS, mitochondrial function, and lipophagy; improved glucose tolerance and reduced AT/liver inflammation.	[65]
IL-4	In vivo (*Fas*-mutant MRL/lpr mice, HFD, cold exposure)	*Fas* mutation increased IL-4, IL-10, UCP1, and tyrosine hydroxylase; promoted M2 macrophages and WAT browning, protecting against HFD-induced obesity.	[66]
IL-4	In vivo (*Leptin*^145E/145E^ mice)	IL-4 reduced body weight, food intake, glucose; modulated hypothalamic neuropeptides (↓AgRP, NPY; ↑POMC); improved insulin–AKT and JAK–STAT signaling; increased UCP1 in WAT.	[67]
IL-4	Ex vivo WAT organotypic culture + in vivo (mice)	IL-4 and IL-13 induced fibrosis-related gene/protein expression via IL-4R and macrophages; partially confirmed in vivo; role in human obesity unclear.	[68]
IL-5	Clinical retrospective study (*n* = 51, severe asthma, anti-IL-5 therapy)	Average BMI decrease of ~1 point; greater reduction in obese subgroup (~1.7 points); confounding factors limit obesity-specific conclusions.	[69]
IL-5	Clinical study (patients with severe asthma, anti-IL-5 therapy)	Similar BMI changes; no consistent differences between agents; effects on body composition unclear.	[70]
IL-5	In vivo (*Il5* Tg/Cd300f^−/−^ mice, HFD)	Increased eosinophils and IL-4 in AT; enhanced M2 macrophages; less weight gain and better glucose tolerance; CD300f deletion amplified these effects.	[70]

Arrows ‘↓’ and ‘↑’ mean ‘decrease’ and ‘increase’ respectively.

**Table 5:** Compounds and modalities modulating Th2 cytokine pathways (IL-4, IL-5, IL-13): approved indications (non-metabolic), evidence in obesity, or metabolic regulation.

Agent/Modality	Target/Pathway	Type	Approved Indications (Non-Metabolic)	Evidence in Obesity/Metabolic Disease	Clinical Trials in Obesity	Ref.
Dupilumab	IL-4Rα (blocks IL-4 and IL-13 signaling)	Antagonist mAb	Atopic dermatitis, eosinophilic asthma, CRSwNP, eosinophilic esophagitis, prurigo nodularis	No interventional obesity trials: metabolic effects not established	None identified	[71]
Tralokinumab	IL-13	Antagonist mAb	Atopic dermatitis	No obesity trials; metabolic outcomes not primary endpoints	None identified	[72]
Lebrikizumab	IL-13	Antagonist mAb	Atopic dermatitis (regional approvals)	No obesity trials reported	None identified	[73]
Mepolizumab	IL-5	Antagonist mAb	Severe eosinophilic asthma; EGPA; HES	Retrospective asthma cohort: mean BMI −1.0 overall; −1.7 in obese subgroup; confounded by corticosteroid changes	None identified	[74]
Reslizumab	IL-5	Antagonist mAb	Severe eosinophilic asthma	Heterogeneous BMI changes in asthma cohorts	None identified	[75]
Benralizumab	IL-5Rα (eosinophil depletion via ADCC)	Antagonist mAb	Severe eosinophilic asthma	No consistent metabolic signal reported	None identified	[75]
IL-4-polarized macrophage exosomes	IL-4-induced M2 programs (miR-21/99a/146b/378a↑, miR-33↓)	Extracellular vesicles (preclinical)	None	In vitro and mice: anti-inflammatory; ↑ browning; improved glucose tolerance	None (preclinical only)	[65]
Pitrakinra (Aerovant)	IL-4/IL-13 (antagonist peptide)	Investigational (discontinued)	None	No obesity data; asthma and eczema studies only	None identified	[76]
Pascolizumab/Anrukinzumab	IL-4 or IL-13	Antagonist mAbs (discontinued)	None	No metabolic evidence	None identified	[77]

Arrows ‘↓’ and ‘↑’ mean ‘decrease’ and ‘increase’ respectively.

## Abbreviations

The following abbreviations are used in this manuscript:

WHO	World Health Organization
T2D	Type 2 Diabetes
CVD	Cardiovascular Diseases
MASLD	Metabolic-associated Steatotic Liver Disease
WAT	White adipose tissue
AT	Adipose Tissue
PWO	People With Obesity
ILC2s	Group 2 Innate Lymphoid Cells
IL	Interleukin
IFN-γ	Interferon-gamma
TNF-α	Tumor Necrosis Factor alpha
BMI	Body Mass Index
GATA3	GATA Binding Protein 3
IgG1/IgE	Immunoglobulin G1/E
HDL-C	High-Density Lipoprotein Cholesterol
SAT	Subcutaneous Adipose Tissue
BAT	Brown Adipose Tissue
VAT	Visceral Adipose Tissue
MCP-1	Monocyte Chemoattractant Protein-1
HGF	Hepatocyte Growth Factor
NFκB	Nuclear Factor Kappa-light-chain-enhancer of Activated B Cells
IK-Kβ	IκB Kinase beta
JNK	c-Jun N-terminal Kinase
NLRP3	NOD-, LRR- and pyrin domain-containing protein 3
IR	Insulin Resistance
EAT	Epicardial Adipose Tissue
PVAT	Perivascular Adipose Tissue
LDL	Low -Density Lipoprotein
HDL	High -dDensity Lipoprotein
LPS	Lipopolysaccharides
TLR	Toll-like receptor
DCs	Dendritic Cells
CD	Cluster of Differentiation (e.g., CD4, CD8, CD25)
CLS	Crown-like Structures
CCL2	C-C Motif Chemokine Ligand 2
LTB4	Leukotriene B4
SV	Stromal Vascular
PRRs	Pattern Recognition Receptors
CCR	C-C Chemokine Receptor
MMEs	Metabolic- Activated Macrophages
ABCA1	ATP-Bbinding Cassette Transporter
TPH-1	Tryptophan Hydrolase 1
UCP-1	Uncoupling Protein 1
HFD	High Fat Diet
NETs	Neutrophil Extracellular Traps
CRAMP	Cathelicidin-Related Antimicrobial Peptide
DIO	Diet-Induced Obesity
SpDCs	Splenic Dendritic Cells
FAO	Fatty Acid Oxidation
Ag	Antigen
Tc1	Type 1 Cytotoxic T cells
PD-1	Programmed cell death 1
TCR	T Cell Receptor
Teff2	T-effector CD4^+^ cells
APCs	Antigen Presenting Cells
IL-4Rα	Interleukin 4 Receptor alpha
FA/FAs	Fatty Acid(s)
STAT	Signal Transducer and Activator of Transcription
PPARα	Peroxisome Proliferator-Activated Receptor alpha
Fgf21	Fibroblast Growth Factor 21
AKT	Protein Kinase B
HSL	Hormone- Sensitive Lipase
cAMP	Cycle adenosine monophosphate
PKA	Protein Kinase A
Tregs	Regulatory T Cells
Th1/Th2/Th17	T helper 1/2/17 cells
AT-EOS	Adipose Tissue Eosinophils
TGF-β	Transforming Growth Factor beta
MIP-1α	Macrophage Inflammatory Protein-1 alpha
AAMs	Alternative Activated Macrophages
ERK	Extracellular signal-regulated kinase
ERR	Estrogen- Related Receptor
GDFIS	Growth Differentiation Factor 15
GFRAL	GDNF Family Receptor alpha-like
